# Effect of dietary inclusions of different types of *Acacia mearnsii* on milk performance and nutrient intake of dairy cows

**DOI:** 10.1016/j.vas.2023.100299

**Published:** 2023-05-29

**Authors:** Lindokuhle C. Mhlongo, Piers Kenyon, Ignatius V. Nsahlai

**Affiliations:** a Animal and Poultry Science, School of Agricultural, Earth and Environmental Sciences, University of KwaZulu-Natal, Private Bag X01, Pietermaritzburg 3209, South Africa; b Ntlangwini Makhoba Farming (PTY) LTD, Makhoba Land, Swartberg, KwaZulu-Natal, 4170, South Africa

**Keywords:** Dry matter intake, Feed efficiency, Milk nutritional quality

## Abstract

This study investigated the effects of including different types of *Acacia mearnsii* (tannin extract and forage) on nutrient intake and milk performance in dairy cattle. Holstein-Friesian x Jersey dairy cows (*n* per Experiment = 24) that had 200 days in milk were selected for this study in a completely randomized study design. This study was conducted under on-farm conditions at Springfontein dairy farm, a farm that lacked a functional bodyweight scale to measure the cow bodyweight and a computer system to register cow parity. Cows were assigned *Acacia mearnsii* tannin extract (ATE) pellets which were added with 0 (0ATE), 0.75 (0.75ATE), 1.5 (1.5ATE) or 3 (3ATE) % ATE in pellets while 0ATE was a commercial protein concentrate (Experiment 1). Cows were assigned *Acacia mearnsii* forage (AMF) at a rate of 0 (0AMF), 5 (5AMF), 15 (15AMF) or 25 (25AMF) % AMF inclusion in corn silage-based diet (Experiment 2). For both Experiments, treatments had six cows each, in which they were adapted (14 d) to diets before data collection (21 d). All AMF inclusions decreased (*P*<0.0001) dry matter intake (DMI), crude protein intake (CPI), neutral detergent fibre intake (NDFI), acid detergent fibre intake (ADFI) and organic matter intake (OMI) at 25AMF. Linear (*P*<0.0001) and quadratic (*P*<0.001) effects were observed on DMI, CPI, NDFI, ADFI and OMI. Inclusions of AMF in corn silage diets affected milk yield, protein yield, lactose yield and milk protein percentage (*P*<0.001). Linear effect was present in milk yield per DMI (*P*<0.0001). In conclusion, the dairy cow diet supplemented with ATE pellets did not have a beneficial effect on the nutrient intakes and milk yield. However, the AMF supplemented in corn silage of the dairy cow diet, increased milk production due to positive effects on nutrient intake, which was favourably influenced from a nutritional point of view.

## 1 Introduction

The dietary incorporation of tannins is used to maximise rumen undegradable protein (RUP) and thus, to enhance milk yield, milk nutritional quality as well as feed efficiency (Mutimura et al., 2018). Feed contains rumen degradable protein (RDP) and RUP but rumen microbes use the former for microbial growth and fermentation efficiency. The RUPs by-pass rumen digestion to get digested in the abomasum and absorbed in the small intestines. Extensive ruminal metabolism of crude protein (CP) decreases RUP in the small intestines (Westwood et al., 2000), which compromises milk production and composition since RUP contributes to milk production as much as RDP (Mikolayunas-Sandrock et al., 2009). Therefore, there is a need to increase rumen RUP of diets using tannins to maximize dairy performance.

Tannins increase RUP by forming a complex with dietary proteins (Ngwa et al., 2002), which is reversed in the abomasum and small intestines at low pH (Animut et al., 2008; Mupangwa et al., 2000). Tannins tend to also bind RDP and fibre which disadvantages rumen nutrient digestibility. Milk performance is a function of nutrient intake as influenced by diet digestibility (Moyo et al., 2018; Mutimura et al., 2018). Milk parameters can benefit from the use of dietary tannins but their adoption is disadvantaged by conflicting nutrient intake and digestibility response to dietary tannins (Matra & Wanapat, 2022). This limitation to the use of dietary tannins in dairy diets is coupled with the non-unilateral effect of dietary tannins on milk yield and composition (Woodward et al., 1999). Due to the tannin-protein or fibre complex presence in tanniferous diets, the dietary tannin content suppresses nutrient intake and digestibility in tannin enriched diets. However, adverse effects of the tannin-protein or fibre complex in tannin sources differ per tannin content (Cardoso-Gutierrez et al., 2021), which explains varying effects of tannin sources on nutrient intake and milk performance (Bhatta et al., 2009). Therefore, tannin sources suppress milk performance by initially decreasing ruminal protein and fibre digestion which leads to decreased nutrient intake and digestibility.

Due to conflicting effects of tannins on dairy performance, dietary tannin sources with low tannin content need exploration to reduce the unpredictable negative effects of tannin sources on nutrient intake and digestibility. Dietary inclusions of low tannin sources could induce a less astringent effect and weak tannin-protein complex which could result in unchanged nutrient intake and digestibility (Yusuf et al., 2017). Low-tannin sources could ensure that the tannin-protein complex is reversible in the small intestines to allow RUP absorption (Animut et al., 2008; Mupangwa et al., 2000). Similarly, low-tannin sources could curtail deleterious effect of tannins on fibre digestibility, subsequently nutrient intake. Therefore, evaluations of low-tannin sources could identify tannin sources to use to supplement dairy diet without compromising nutrient intake and digestibility, and subsequently milk performance (Mkhize et al., 2014).

The dietary inclusion of *Acacia mearnsii* tannin extract (ATE) might enhance milk performance and nutrient intake (Avila et al., 2020; Griffiths et al., 2013) but its on-farm effect on these variables is premature. The dietary inclusion of this tannin source has a detrimental effect on both milk performance and intake at excessive dietary inclusion rates (Grainger et al., 2009). The ATE dietary inclusion has also been reported for increased intake and decreased milk parameters (Williams et al., 2020). Conflicting ATE effect on milk performance could be ascribed to varying astringency in tannin sources. High tannin astringency reduces nutrient intake and digestibility by complexing nutrients, fibre and CP. However, the ATE supplementation through pellets could coat the negative effect of ATE inclusions on nutrient intake and digestibility (Griffiths et al., 2013). Nutrient intake has remained unchanged and nutrient digestibility has increased when coated ATE are fed (Adejoro et al., 2019). Therefore, more research needs to expand on the effect of ATE pellets on nutrient intake and digestibility. Additionally, *Acacia mearnsii* forage (AMF) is a low tannin version of ATE that has not been studied in detail for its on-farm effect on milk performance and feed intake.

Under on-farm conditions, more studies need to evaluate the same tannin source in various physical presentations and dietary inclusions to reduce negative effects of tannin sources on nutrient intake and milk parameters. The objectives of this study were to investigate the on-farm impact of different dietary inclusions of ATE or AMF on nutrient intake and milk performance. This study hypothesised that the increased dietary inclusions of ATE in pellets or AMF in corn silage would decrease nutrient intake and milk performance due to tannin's negative effect on nutrient intake and digestibility.

## 2 Materials and methods

### 2.1 Study site, animals, treatments, and management

These Experiments were conducted at the Springfontein dairy farm in Kokstad, KwaZulu-Natal, South Africa (30°15ꞌ52.0ꞌꞌS, 29°10ꞌ15.0ꞌꞌE S and 1450 m altitude). The KZN-DARD Research Committee (ref: 12/11/1/15 (2387JD)) and UKZN Animal Research Ethics Committee (ref: AREC/00,003,470/2021) approved this study.

### 2.2 Experiment 1

In a completely randomized design, a total of twenty-four cows were divided into four groups of six cows each. Cows selected for this study were a cross-breed of Holstein-Friesian and Jersey cows that had ⁓200 days in milk (199.5 ± 5.12 days in milk±SD). By randomly drawing a treatment from the bag, each cow was assigned to treatments. The treatments were 0 (0ATE), 0.75 (0.75ATE), 1.5 (1.5ATE) or 3 (3ATE) % inclusion of ATE in pellets (Tseu et al., 2020)(Tables 1 and 2). The 0ATE pellets were a 15% CP commercial concentrate (AFGRI, Pietermaritzburg, South Africa) that was fed to the herd at the study site during milking, while the ATE pellets were prepared by PSP Traders (Kokstad, South Africa). The individual ingredients of 0ATE were withheld by the producer; hence they do not appear in Table 1. The ATE had a purity of 68% and its purity for condensed tannins was determined using the butanol-HCl method (Mimosa Central Cooperative Ltd, Pietermaritzburg, South Africa)*.* The cows used in this study were from a farm that lacked records on parity and body weight of each cow due to defective computer system and bodyweight scale, respectively. Each cow was allocated 2 kg (as-fed) of experimental pellets per morning and afternoon milking session. Cows were kept with the non-experimental herd and fed pasture consisting of perennial ryegrass (*Lollium perrene*) + white clover (*Trifolium pratense*) during the day between milking sessions at 5:00 and 14:00 h. Cows were fed corn silage and soyabean silage *ad libitum* during the night separately from the non-experimental herd of the farm. The study lasted for 35 days whereby cows were adapted to the diets for 14 days and used for data collection for 21 days (Griffiths et al., 2013). Cows were dipped for mastitis before and after milking. For each treatment group, cows were marked using animal spray paint for identification.

**Table 1:** Composition of experimental pellets.

Item (kg)	ATE (%)		
	3	1.5	0.75
S/Run	498	498	498
Hominy Chop	275	275	275
Bran	100	100	100
Molasses Liquid	50	50	50
Soya Oil Cake	30	30	30
Urea	15	15	15
Lime Powder	12.5	12.5	12.5
Salt	10	10	10
MCP 21	5	5	5
Px 10,149 Dairy (5 Kg)	5	5	5
ATE	44	22	11

**Table 2:** Chemical composition (g/kg) of the basal feeds and experimental pellets.

Item (g/kg)	DM	Ash	EE	ADF	NDF	CP
Corn silage	964.4	39	28.5	220	396	65
Perennial ryegrass + White clover	941	150	18.5	222	356	217
ATE pellets	916.3	64	26.5	48.9	163.8	145.3
Control pellets	980	88.5	22.5	110.1	280.7	150
Soyabean silage	573	193	25	281	345	11.5

DM, dry matter, EE, ether extract, ADF, acid detergent fibre, NDF, neutral detergent fibre, CP, crude protein. The chemical compositions were derived from the feed composition report of the farm.

### 2.3 Experiment 2

Twenty-four crossbreed cows (Holstein-Friesian x Jersey) of four groups of six cows each with ⁓200 days in milk (211.46±16.41 days in milk±SD) were selected for this study. Parity could not be used as part of the study design as the farm did not have accurate records on the parity of the herd. The digital scale for measuring bodyweight was also not functional at the farm which resulted in the lack of bodyweight measurements of the cows in the present study. The study was a completely randomized design. The cows were assigned to each of four treatments by randomly drawing the treatments at random from a bag that contained each treatment. The treatments were 0 (0AMF), 5 (5AMF), 15 (15AMF) or 25 (25AMF) % inclusion of AMF in corn silage (Tables 2 and 3), each treatment had six cows. The trial lasted for 35 days which consisted of a 14 day adaptation period and a 21 day data collection period (Griffiths et al., 2013). For each treatment group, cows were marked using animal spray paint for identification.

**Table 3:** Chemical composition of experimental feeds.

Feeds	Chemical composition (g/kg)							
	DM	Ash	EE	ADF	NDF	CP	ADIN	CT
Lucerne hay	967.3	108	28.7	304.2	406.4	119.6	9.3	–
AMF	879.7	44.7	31.8	658.3	753.3	157	16.5	31
0AMF	964.6	56.5	25.8	339.8	607.4	81	6.6	–
Veld hay	940.4	63.3	14.8	522.2	844.1	69.1	5.4	–
Ryegrass + white clover	964.5	121.8	18.5	293.1	536.5	219.1	12.6	–
5AMF	966.8	201	20.1	289.6	476.8	67.2	–	1.56
15AMF	969.2	49.4	24.4	37.73	615.1	75.4	–	4.65
25AMF	964	46	29.3	454.7	641.3	97.9	–	7.75

0AMF, 0% AMF inclusion in corn silage (CS); 5AMF, 5% AMF inclusion in CS;15AMF, 15% AMF inclusion in CS; 25AMF, 25% AMF inclusion in CS ; DM, dry matter; EE; ether extract, ADF, acid detergent fibre; NDF; neutral detergent fibre and CP; crude protein, CT, Condensed tannins and ADIN, Acid detergent insoluble nitrogen.

Cows were fed treatments after the afternoon milking session at 14:00 pm for 2 h inside locked, non-sheltered and wooden individual pens (2.4 m, length, 3.12 m width and 1.03 m height). Barb wires and iron standards were used to create gates for each pen. Each pen contained fresh water in plastic tubs (20 L). Plastic feeders were attached on the frontal part of each pen between the slatted poles (51 cm apart) built on either side of each pen for each cow to fit their head to access feed. A 60-point rotary De-Laval (E100, DeLaval, Pinetown, South Africa) milking equipment was used for milking cows.

Fresh corn silage was collected from the corn silage pit daily and mixed with weighed AMF inclusions of this study using a portable scale. The AMF and corn silage mixture was stored in labelled feed bags, shaken for mixing and fed to each cow daily. Whole plant of AMF was milled using a woodchipper (Tomcat 150 CDE Wood-Chipper, Worcester, South Africa) and dried under shed for seven days before use. A cell phone timer was used to measure the 2-hour feeding time. Cows were released from pens to have gracious feeding on Lucerne (*Medicago sativa*) that had been stored for a year and veld hay overnight. Lucerne used at the farm consisted mostly of stalks than leaves and potentially affected its chemical composition. Cows grazed white clover and perennial ryegrass (*Lolium perenne*) between the morning (5:00 am) and afternoon (14:00 pm) milking session. Cows were teat dipped on each quarter for mastitis at the end of each milking session and offered a custom mix concentrate (AFGRI, Pietermaritzburg, South Africa) with 15% CP content per milking session. One cow in the control treatment aborted and was removed from the assigned group at the beginning of the data collection period.

### 2.4 Data collection

For both Experiments, the milk yield of each cow was recorded daily from the screen of each milking unit per milking session at 4:00 am and 14:00 pm. Only for Experiment 2, milk samples were collected once per week under the data collection period during the morning and the afternoon milking sessions by hand or milk bottle samplers. Milk was sampled (200 ml) using bottle samplers or hand milking each quarter per cow at the end of milking when a bottle sampler malfunctioned. Milk bottle samplers were sanitized for each sampling period and attached to milk lines to ensure the sampling of milk from every litre per cow per treatment. Milk samples were shaken, emptied into labelled vials and preserved with a drop of bronopol. Labelled milk vials containing milk samples (90 ml) were subjected to immediate storage in a cooler box containing ice blocks during milk sampling, refrigerated at the end of milk sampling and transported to the laboratory (Greenhills laboratories, Hilton, South Africa) the following day for milk composition analysis. Two of the six cows assigned to 25AMF were not sampled for milk during the first week of data collection due to being medicated for mastitis but were kept for the duration of the trial. For both Experimental 1 and 2, feed samples were collected weekly by manual grabs during the data collection period using labelled zip lock bags, manually pooled into one sample and refrigerated pending analysis. Pasture was sampled diagonally in grazing camps. Bales were sampled by sampling the top and bottom part of each bale.

### 2.5 Chemical analyses

Feed samples were forced air-oven-dried (60°C for 72 h) for preservation, ground (2 mm sieve), packed in labelled zip lock bags and sent to Cedara analytical laboratory (Hilton, South Africa) for proximate analysis. Feed samples were analysed for nitrogen content (ID 968.06) using the Leco Truspec nitrogen analyser and nitrogen content was multiplied by 6.25 to estimate Crude protein (CP) (Leco FP200, LECO, Pretoria, South Africa). Dry matter (DM) (ID 934.01) and ash (ID 942.05) were analysed using the AOAC method (AOAC, 2000). ANKOM 220 Fibre analyser (ANKOM Technology, Fairport, NY, USA) was used to determine acid detergent fibre (ADF) and neutral detergent fibre (NDF) using the filter bag technique (ANKOM Technology, 2011). The Soxhlet Buchi 810 Fat analyser (Soxhlet Buchi, Flawil, Switzerland) was used to estimate the ether extract content.

Only for Experiment 2, milk samples were labelled according to treatments and stored in a cooler box during transportation to the laboratory (Greenhills laboratories, Hilton, South Africa) for milk quality analyses. The ATE and AMF samples were sent to MIMOSA extract company (NTE house, Redlands Estate, Pietermaritzburg 3201, South Africa). Tannin content analysis using the Society of Leather Technologists and Chemists (SLTC) method (SLTC, 1965); where briefly 200 g of feed was heated under reflux with water (1 litre and for 5 h) to extract tannins. The extract liquor was then filtered off and the process was done twice to extract as many tannins as possible, the extract liquor was concentrated to dryness (70 °C) under vacuum to a brown liquid. Hide powder was mixed with the brown liquid and stirred. The mixture was left to stand and filtered. The filtrate was evaporated to determine non-tannins while the tannin content was determined as the difference between soluble solids and non-tannins.

### 2.6 Mathematical calculations and statistical analyses

For both experiments, daily feed intake was measured by weighing feed refusals after the animals had finished feeding and subtracting feed refused from the weighed feed offered. For both Experiments, acid detergent fibre intake (ADFI), neutral detergent fibre intake (NDFI), organic matter intake (OMI) and crude protein intake (CPI) were calculated by multiplying the percentage of each chemical component by intake adjusted for dry matter intake (DMI). DMI was dertemined by multiplying feed intake with the DM percentage. The efficiency of milk production was calculated by dividing milk yield with DMI. Milk fat, protein, and lactose yield were calculated by multiplying their respective content percentages with milk yield.

The generalised linear model procedure of SAS (9.4) was used to determine the effect of dietary inclusions of ATE or AMF on milk production and nutrient intake in dairy cows. The data were analysed using the following model: $\;Y_{i}=\mu +\alpha_{i}+\varepsilon_{i}$, where$\;Y_{i}$ is the dependant variable, $\alpha_{i}$ is the treatment effect. Tukey test was used to test the differences between treatment means. The results were reported as least square means and standard error of means (SEM). The orthogonal polynomial contrast were used to determine the linear and quadratic effects to identify the relationship between ATE or AMF inclusions and milk variables or nutrient intake. Statistical significance was declared at *P*<0.05.

## 3 Results

Table 4 shows that the effect of the dietary inclusions of ATE on milk yield and nutrient intakes of DM, OM, CP, NDF and ADF was not significant (*P*>0.05). The linear or quadratic effects were not significant (*P*>0.05) as well.

**Table 4:** Effect of different dietary inclusions of ATE in pellets on milk yield and intakes in dairy cows (Experiment 1).

Items	0ATE	0.75ATE	1.5ATE	3ATE	SEM	Significance		
						P-value	Linear	Quadratic
Milk yield, kg/d	14.22	15.7	12.64	14.65	1.67	0.63	0.82	0.88
DMI, kg/d	3.67	3.63	3.67	3.67	0.02	0.413	0.66	0.33
OMI, kg/d	3.43	3.4	3.43	3.43	0.02	0.413	0.66	0.33
CPI, kg/d	0.53	0.53	0.53	0.53	0.003	0.413	0.66	0.33
NDFI, kg/d	0.60	0.59	0.60	0.60	0.003	0.413	0.66	0.33
ADFI, kg/d	0.18	0.18	0.18	0.18	0.0008	0.413	0.66	0.33

ATE, *Acacia mearnsii* tannin extract; 0ATE, 0% ATE inclusion in pellets; 0.75ATE, 0.75% ATE inclusion in pellets; 1.5ATE, 1.5% ATE inclusion in pellets; 3ATE, 3% ATE inclusion in pellets; DMI, dry matter intake; ADFI, acid detergent fibre intake; NDFI; neutral detergent fibre intake; OMI, organic matter intake; CPI, crude protein intake; SEM, Standard error of means.

Table 5 shows that AMF inclusions in corn silage diets negatively affected intake (*P*<0.0001) of DM, OM, ADF, NDF and CP compared to 0AMF. There were significant linear (*P*<0.0001) or quadratic (*P*<0.01) effects on nutrient intake.

**Table 5:** Effect of different dietary inclusions of AMF in corn silage on nutrient intakes in dairy cows (Experiment 2).

Intake (kg/d)	0AMF	5AMF	15AMF	25AMF	SEM	Significance		
						P-value	Linear	Quadratic
DM	4.90^a^	4.47^a^	4.36^a^	3.13^b^	0.15	<0.0001	<0.0001	0.0078
CP	0.40^a^	0.39^a^	0.41^a^	0.30^b^	0.01	<0.0001	<0.0001	0.0006
OM	4.62^a^	4.28^a^	4.11^a^	2.93^b^	0.14	<0.0001	<0.0001	0.0032
ADF	1.65^a^	1.62^a^	1.68^a^	1.22^b^	0.06	<0.0001	<0.0001	0.0008
NDF	2.95^a^	2.80^a^	2.74^a^	1.99^b^	0.09	<0.0001	<0.0001	0.0014

AMF, *Acacia mearnsii* forage; 0AMF, 0% AMF inclusion in corn silage; 5AMF, 5% AMF inclusion in corn silage; 15AMF, 15% AMF inclusion in corn silage; 25AMF, 25% AMF inclusion in corn silage, DM; dry matter, ADF; acid detergent fibre, NDF; neutral detergent fibre, OM; organic matter, CP; crude protein; SEM, Standard error of means; a,b Means within a row with no common superscript differ (*P*< 0.05).

Table 6 shows that AMF inclusions in corn silage affected milk yield (*P*<0.0001), lactose yield (*P*<0.0001), milk protein yield (*P*<0.01), milk protein percentage (*P*<0.01) and feed efficiency (milk yield/DMI)(*P*<0.0001). Linear or quadratic effects did not affect milk composition and MUN (*P*>0.05). The linear effect affected (*P*<0.0001) feed efficiency.

**Table 6:** The effect of different dietary AMF inclusions in corn silage on milk yield and composition in dairy cows (Experiment 2).

Items	0AMF		5AMF		15AMF		25AMF	SEM	Significance		
									P-value	Linear	Quadratic
Milk quantity (kg/d)											
Milk yield	14.12^b^	12.05^c^		16.67^a^		13.85^bc^		0.51	<0.00001	0.0677	0.3513
Fat yield	0.74	0.70		0.78		0.66		0.05	0.256	0.4907	0.3552
Protein yield	0.53^ab^	0.50^b^		0.60^a^		0.53^b^		0.02	0.004	0.4336	0.2135
Lactose yield	0.61^b^	0.51^c^		0.74^a^		0.60^bc^		0.03	<0.0001	0.0962	0.3526
Milk quality (%)											
Fat	5.34	5.70		4.70		4.74		0.33	0.075	0.0559	0.7048
Protein	3.83^ab^	4.14^a^		3.60^b^		3.78^b^		0.10	0.004	0.0560	0.5417
Lactose	4.34	4.20		4.41		4.32		0.07	0.122	0.6241	0.8235
MUN (mg/100 g)	12.38	10.36		11.90		10.79		1.42	0.703	0.7097	0.6492
Feed efficiency											
Milk/DMI	2.85^bc^	2.67^c^		3.96^ba^		4.95^a^		0.33	<0.0001	<0.0001	0.0728

AMF, *Acacia mearnsii* forage; 0AMF, 0% AMF inclusion in corn silage; 5AMF, 5% AMF inclusion in corn silage; 15AMF, 15% AMF inclusion in corn silage; 25AMF, 25% AMF inclusion in corn silage; MUN; Milk urea nitrogen, SEM; Standard error of means; a,b Means within a row with no common superscript differ (*P*< 0.05).

## 4 Discussion

The present study's observation of the absence of the effect ATE inclusion in pellets on nutrient intakes, milk production and milk quality corroborates the findings of Kapp-Bitter et al. (2020). The small particle size of pellets enhance nutrient intake by increasing passage rate of digesta in the rumen. Rumen microbes easily access substrates in small particle sized feeds which allows more nutrient intake by increasing ruminal digestion and passage rate of feeds (Zhong et al., 2018). In the current study, it was expected that ATE inclusions in pellets would affect nutrient intake due to the astringent effect of tannins. The astringent effect of tannins reduces nutrient intake rate (Lima et al., 2019) by increasing eating, chewing and ruminating time as dietary tannins increase (Rigueira et al., 2021; Tseu et al., 2020). However, ATE inclusions in pellets of the present study must have been insufficient to influence the feeding behaviour of the cows (Oliveira et al., 2023) or had their effect masked by the physical form of ATE pellets (Denninger et al., 2020; Focant et al., 2019). Therefore, the current study's observation of reduced nutrient intake from non-pelleted AMF inclusions may have been due to feed sorting as induced by AMF astringency (Oliveira et al., 2023).

The present study's demonstration of decreased nutrient intake by AMF inclusions corroborate previous results on non-pellet diets with tannins (Dschaak et al., 2011; Muamba et al., 2014; Ngwa et al., 2002). In tanniferous diets, nutrient intake is negatively affected due to the presence of astringent effect in condensed tannins (Naumann et al., 2017). Condensed tannins bind to salivary proteins which induce a feed astringency and this effect is positively correlated to dietary tannin inclusions (Lamy et al., 2011). In the present study, the nutrient intakes in 25AMF must have decreased due to increased astringent effect. To maintain nutrient intake at 5AMF and 15AMF, cows must have adopted the ruminant's strategy of masking the negative effect of the tannin-salivary proteins complex by secreting more salivary proteins (Alonso-Díaz et al., 2012; Lamy et al., 2011). However, 25AMF suppression of nutrient intake must have been due to the salivary protein reaction tannins being insufficient to coat the tannin astringent effect (Salem et al., 2013). The effect of the tannin-salivary protein complex on nutrient intake also depends on the condensed tannin molecular weight (Saminathan et al., 2014), which potentiates for tannin sources of high molecular weight to depress nutrient intake similarly at moderate inclusions and adversely at high dietary inclusions. The nutrient intake depression by AMF suggests that coating of its tannins, which has shown positive results on nutrient intake, could reduce its adverse effect on nutrient intake shown by 25AMF (Adejoro et al., 2019).

To prevent the nutrient intake depression, the maximum recommended dietary inclusion of tannins in diets (DM) is five per cent (Piñeiro-Vázquez et al., 2015). Five per cent dietary DM inclusions of tannins could be the maximum point at which the molecular weight and tannin-salivary proteins effects on nutrient intake remain bearable. Less than five per cent DM inclusions of tannins lacks a negative effect on nutrient intake and digestibility (Avila et al., 2020; Pedraza-Beltrán et al., 2012). Milk parameters also remain unchanged by diets with less than five per cent DM inclusion of tannins due to unchanged nutrient intake and digestibility (Avila et al., 2020; Pedraza-Beltrán et al., 2012). Tannins bind proteins and fibre to form a tannin-protein or fibre complex which reduces ruminal fibre and protein digestibility (Yogianto et al., 2014). Reduced fibre and protein digestibility translates into firstly compromised nutrient intake and secondly reduced microbial proteins which are required to enhance milk parameters. In the current study, the observed oscillating effect of AMF inclusions on milk yield, milk protein yield and milk protein percentage are similar to those of Toral et al. (2013) and could be attributed to the negative influence of AMF tannins on nutrient intake and digestibility. The results of the present study indicate that increased milk yield between 5AMF and 15AMF might have been due to increased nutrient intake between the two treatments, which is in contrast to results obtained with the ATE inclusion.

In the current study, the shown 5AMF, 15AMF and 25AMF effect on nutrient intake or milk performance relative to 0AMF could be due to indigestible fibre content in addition to the tannin content, which was less than five per cent DM (3.1%). The AMF is part of Acacia species and these species contain high amounts of acid detergent lignin (ADL) which hinder rumen fermentation (Rubanza et al., 2005). Treating AMF with exogenous enzyme might improve their utilization by dairy cows as it has been previously shown that exogenous fibrolytic enzyme increase milk parameters (Gado et al., 2009; Golder et al., 2019). Fibrolytic enzymes enhance DM digestibility (Yang et al., 2022) by increasing DM, OM and CP, and decreasing NDF and ADL (Kumar et al., 2021).

## 5 Conclusions

This study indicated that ATE inclusions in pellets did not affect milk yield and nutrient intake. However, it showed that AMF inclusions in corn silage decreased nutrient intakes, milk yield and milk protein percentage. The AMF inclusions should not exceed 15% in the diet to prevent the compromise of nutrient intake, milk yield and milk protein percentage in dairy cows. On-farm studies based on the dietary inclusion of exogenous fibrolytic enzymes in AMF on milk parameters and nutrient intake and digestibility are recommended to enhance the understanding of AMF effect on dairy performance.

## CRediT authorship contribution statement

L.C.M., P.K. and I.V.N. conceived the paper. L.C.M. and P.K. acquired funding. L.C.M. collected and analysed the data and wrote the paper. All authors contributed valuable comments and gave approval for publication.

## Compliance with ethical standards

KZN-DARD Research Committee (ref: 12/11/1/15 (2387JD)) and UKZN Animal Research Ethics Committee (ref: AREC/00,003,470/2021) approved this study.

## Ethical statement for solid state ionics

Hereby, I /insert author name/ consciously assure that for the manuscript /insert title/ the following is fulfilled:

1) This material is the authors' own original work, which has not been previously published elsewhere.
2) The paper is not currently being considered for publication elsewhere.
3) The paper reflects the authors' own research and analysis in a truthful and complete manner.
4) The paper properly credits the meaningful contributions of co-authors and co-researchers.
5) The results are appropriately placed in the context of prior and existing research.
6) All sources used are properly disclosed (correct citation). Literally copying of text must be indicated as such by using quotation marks and giving proper reference.
7) All authors have been personally and actively involved in substantial work leading to the paper, and will take public responsibility for its content.

The violation of the Ethical Statement rules may result in severe consequences.

To verify originality, your article may be checked by the originality detection software iThenticate. See also http://www.elsevier.com/editors/plagdetect.

I agree with the above statements and declare that this submission follows the policies of Solid State Ionics as outlined in the Guide for Authors and in the Ethical Statement.

## Declaration of Competing Interest

The authors declare no conflict of financial interest interest or personal relationships that could have appeared to influence the work reported in this paper.

This study was supported by Council for Scientific and Industrial Research-Interbursary, HWSETA postgraduate bursary and Inyosi Empowerment under the Nestlé Makhoba Youth Employment Services (YES) Program.
